# Bats expand their vocal range by recruiting different laryngeal structures for echolocation and social communication

**DOI:** 10.1371/journal.pbio.3001881

**Published:** 2022-11-29

**Authors:** Jonas Håkansson, Cathrine Mikkelsen, Lasse Jakobsen, Coen P. H. Elemans

**Affiliations:** Sound Communication and Behavior Group, Department of Biology, University of Southern Denmark, Odense M, Denmark; University of Zürich, SWITZERLAND

## Abstract

Echolocating bats produce very diverse vocal signals for echolocation and social communication that span an impressive frequency range of 1 to 120 kHz or 7 octaves. This tremendous vocal range is unparalleled in mammalian sound production and thought to be produced by specialized laryngeal vocal membranes on top of vocal folds. However, their function in vocal production remains untested. By filming vocal membranes in excised bat larynges (*Myotis daubentonii*) in vitro with ultra-high-speed video (up to 250,000 fps) and using deep learning networks to extract their motion, we provide the first direct observations that vocal membranes exhibit flow-induced self-sustained vibrations to produce 10 to 95 kHz echolocation and social communication calls in bats. The vocal membranes achieve the highest fundamental frequencies (*f*_*o*_*’s*) of any mammal, but their vocal range is with 3 to 4 octaves comparable to most mammals. We evaluate the currently outstanding hypotheses for vocal membrane function and propose that most laryngeal adaptations in echolocating bats result from selection for producing high-frequency, rapid echolocation calls to catch fast-moving prey. Furthermore, we show that bats extend their lower vocal range by recruiting their ventricular folds—as in death metal growls—that vibrate at distinctly lower frequencies of 1 to 5 kHz for producing agonistic social calls. The different selection pressures for echolocation and social communication facilitated the evolution of separate laryngeal structures that together vastly expanded the vocal range in bats.

## Abstract 2

Echolocating bats produce very diverse vocal signals for echolocation and social communication that span an impressive seven octaves. This study uses high-speed video and audio to reveal that distinct laryngeal structures underlie echolocation and agonistic social calls in bats.

## Introduction

The evolution of powered flight, echolocation, and subsequent fast buzzing allows bats to hunt and capture fast-moving airborne prey and thereby exploit the riches of the night: flying insects [1,2]. To detect small prey, biosonar signals need to contain high frequencies to provide efficient acoustic reflection and high bandwidth to provide high localization accuracy and spatial resolution [3]. Echolocation thus selects for increased fundamental frequency, *f*_o_ and expansion of the *f*_o_ range, and many species of bats (FM bats) produce precisely timed, frequency-modulated echolocation calls that sweep in *f*_o_ from as high as 125 kHz down to approximately 10 kHz in calls of only 1 to 2 ms duration [3–5]. Some species have calls with *f*_o_ up to 250 kHz [6], and, as such, bats produce the highest known voiced *f*_o_ of all mammals. In addition, many bat species produce social communication calls [7] of which some extend their *f*_o_ range further down to 1 kHz [8,9]. Thus, bats can produce very diverse signals that span an impressive *f*_o_ range of 1 to 120 kHz or 6 to 7 octaves, while humans and other mammals typically only produce 3 octaves and in exceptional cases 4 to 5 [10]. The tremendous vocal range of bats is unparalleled in mammalian sound production, but how bats achieve this remains unknown.

Bat calls are produced laryngeally as in most mammals [11,12], but in bats, the vocal folds exhibit several adaptations compared to the generalized mammalian vocal fold, likely associated with echolocation requirements [13–16]. First, the paired vocal folds end in 6 to 10 micrometer thin, apical vocal membranes [15]. Such membranes have been reported in bats, cats, and nonhuman primates and have been suggested to act as low-mass oscillators that can vibrate almost independently of the vocal fold proper, and thereby support the production of high-frequency vocalizations [17–19]. In marmosets, the first direct observation of vocal membrane vibration showed that they can indeed vibrate at frequencies up to 9 kHz [19]. However, in bats, we lack direct observation of vocal membrane vibration and their function in vocal production remains untested. Second, a second smaller apical membrane points downwards from the ventricular folds, the ventricular membrane [15]. In general, the ventricular folds have received very little attention in comparative bioacoustics, and while their altered geometry in bats suggests function, this remains untested.

A combination of different laryngeal structures may serve to facilitate the tremendous *f*_o_ range and different call types bats produce. Indeed, other mammals, such as marmosets can switch from vocal fold to vocal membrane vibration over postnatal development [19]. In humans, ventricular folds play a role in several low-frequency forms of singing, such as death metal grunting and Tuvan throat singing, where they can touch the vocal fold and increase the mass of the oscillating structures [20]. This results in a much lower *f*_o_ than can be achieved by the vocal folds alone [20,21]. Additionally, human vocal folds can exhibit different oscillation regimes in the different voice registers, such as vocal fry, chest, and falsetto that expand the vocal range [22,23]. In an excised bat larynx preparation, abrupt changes in acoustics were attributed to such register jumps [11]. However, no direct evidence exists as to if and how laryngeal and ventricular structures can vibrate to produce sound in bats due to challenges of imaging the vocal folds in vivo at the extremely high speeds required.

Here, we test the hypothesis that specialization of different laryngeal structures supports the extreme frequency range of FM bats. We test this hypothesis in Daubenton’s bats (*Myotis daubentonii*) that have an extreme 77 octave *f*_o_ range from 1 to 95 kHz [9,24].

## Results

**Fig 1:**
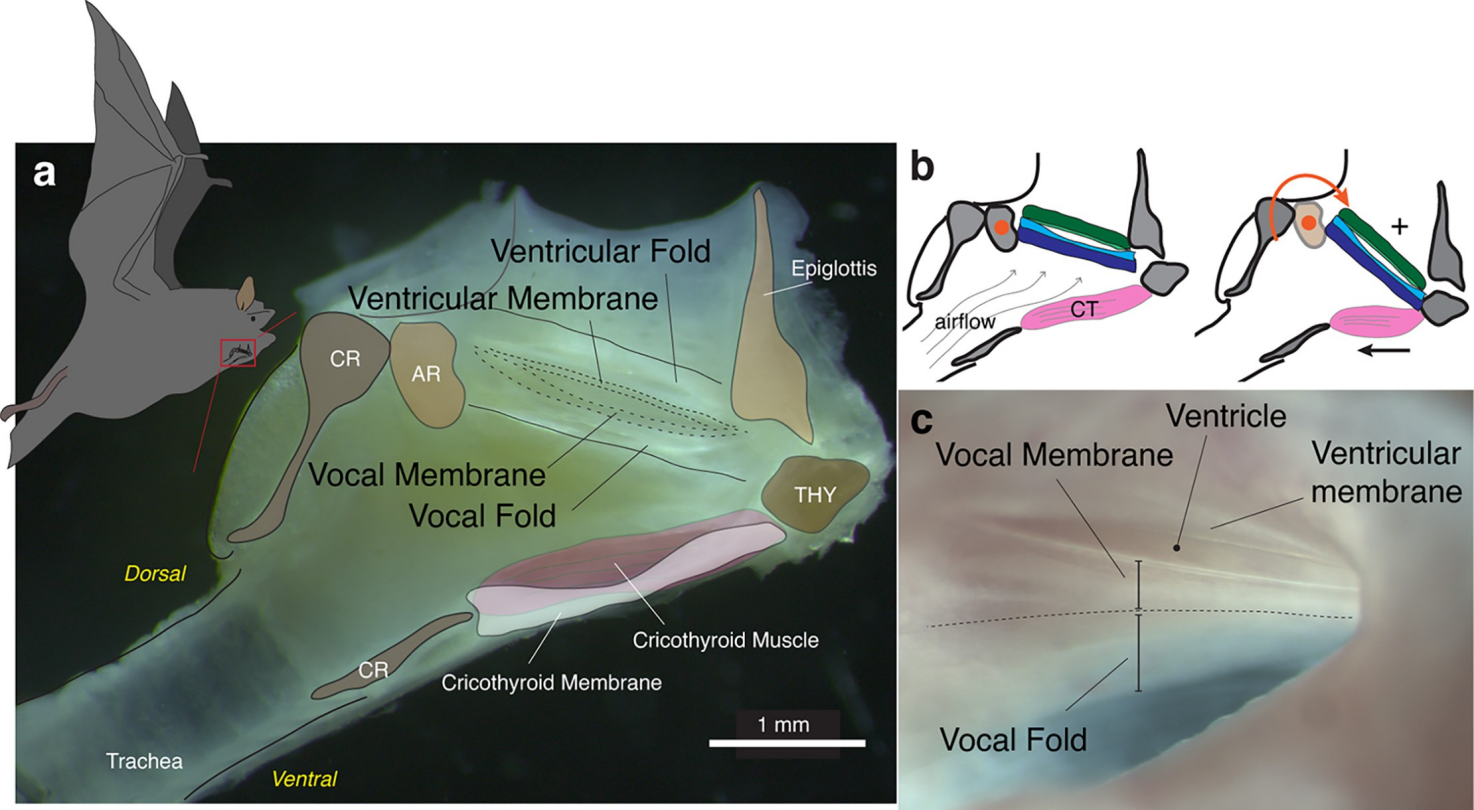
Specialized anatomy of the microchiropteran bat larynx. (a) Sagittal cross-section of the larynx of *M*. *daubentonii*. The paired vocal and ventricular folds with apical membranes are suspended between arytenoids and thyroid. (b) The mechanism of sound production is hypothesized to be airflow-induced vibration of the vocal membranes (left). Contraction of the cricothyroid muscle rotates the thyroid around the cricoarytenoid joint (right) and thereby elongates and increases tension in the vocal folds and membranes (+), which in turn increases the *f*_*o*_. (c) Detail of left ventricular and vocal folds with thin apical membranes. AR, arythenoid; CR, cricoid; THY, thyroid cartilage.

While all echolocating bats are assumed to have apical vocal membranes on their vocal folds, it is only established in <10 out of approximately 1,100 species [15,16]. We confirmed the presence of vocal membranes in *M*. *daubentonii* by extracting the fresh larynx from 5 individuals. Visual inspection through the narrow epiglottic opening showed the presence of vocal and ventricular folds separated by the laryngeal ventricle, aka the ventricle of Morgagni, in all individuals (Fig 1). We confirmed the presence of thin apical vocal membranes extending cranial on the vocal fold and caudal on the ventricular fold. The overall laryngeal anatomy includes several unique adaptations compared to a generalized mammalian larynx as described for *Eptesicus fuscus* [15,16,25]; (1) a hypertrophied laryngeal musculature, particularly the cricothyroid muscle; (2) a large cricothyroid membrane; and (3) calcified cricoid and thyroid cartilages.

**Fig 2:**
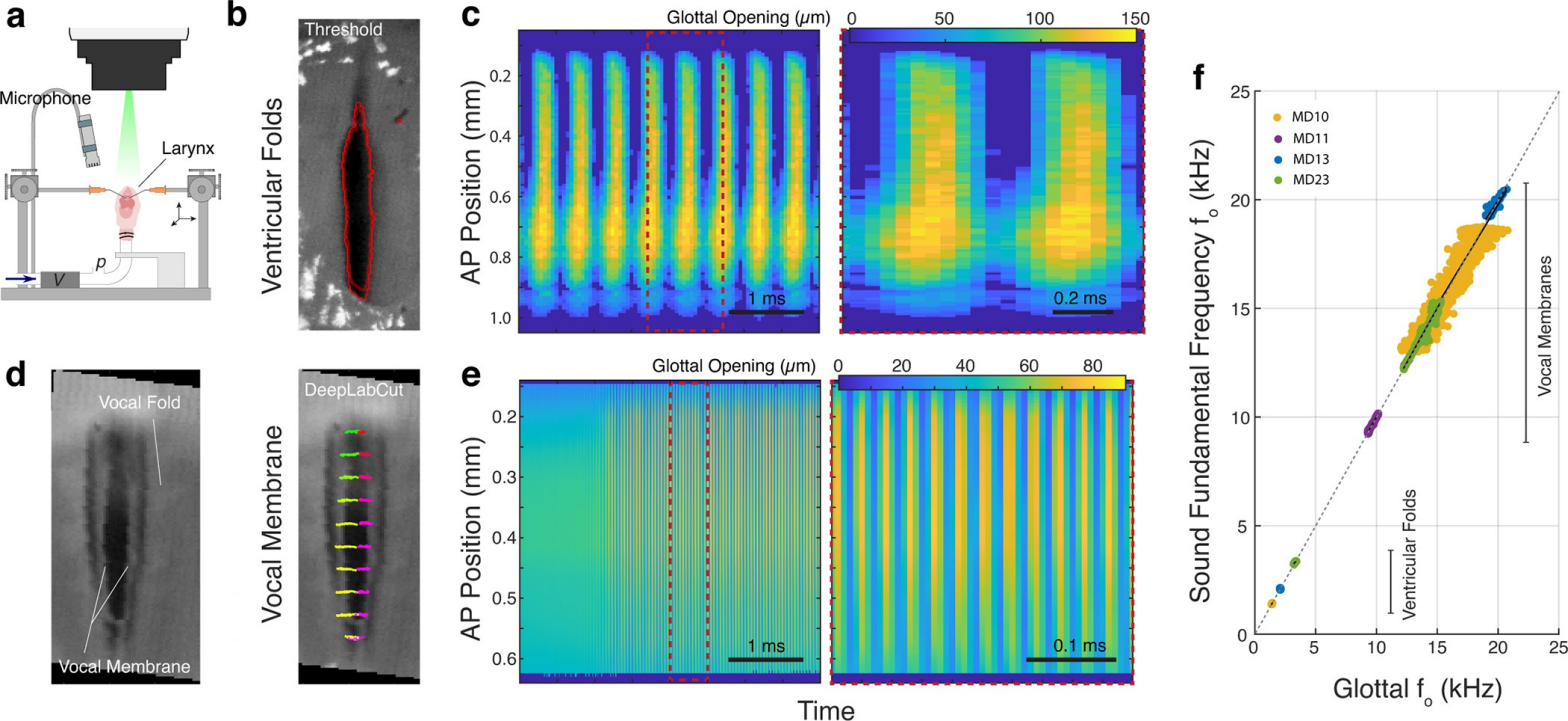
Ventricular folds and vocal membranes produce sound and in different frequency ranges. (a) Sketch of excised larynx setup (see Materials and methods). (b) Video still of ventricular fold vibration filmed at 25,000 fps with annotated glottal boundary (red) using gray threshold. (c) Glottovibrogram of ventricular fold vibration (left) and detail (right) color coded for glottal width. (d) Video still of vocal membrane filmed at 125,000 fps. The edge of the vocal membrane was detected using the deep learning network (DeepLabCut). Colored traces depict movement of 10 locations along the vocal membranes that were detected along the vocal membrane tip. (e) Glottovibrogram of vocal membrane vibration (left) and detail (right). (f) The *f*_*o*_ of vibration equals that of produced sound (S1 Table) showing that the structures generate the sound, but in distinct frequency ranges. Colors indicate individuals; dotted gray line is the identity line. The data underlying c, e, and f can be found in S1, S2, and S3 Data files.

We next mounted these larynges in an excised larynx setup (Fig 2A, see Materials and methods) [19,26–28]. After approximation of the ventricular folds with micromanipulators, increasing the bronchial pressure induced self-sustained vibration of the ventricular folds (Fig 2B and 2C and S1 Movie) in 3 out of 3 individuals. We could not see past the ventricular folds and could thus not observe if the vocal folds and vocal membranes also vibrated. The glottal opening was darker than the ventricular folds and the glottal opening dynamics could reliably be extracted with a simple threshold method (Fig 2B). The *f*_o_ of these oscillations laid on the identity line of the sound and vibration *f*_o_ (Fig 2F), strongly suggesting this vibration caused the sound pressure signal. The *f*_o_ of ventricular fold vibrations ranged between 1 and 3 kHz.

To allow visual access to the vocal folds and vocal membranes, we removed the ventricular folds by carefully cutting through the ventricle of Morgagni. After approximating the vocal folds, increasing the bronchial pressure induced self-sustained oscillations of the vocal membranes in 4 out of 4 individuals. To ensure accurate capture of the fast motion of the vocal membranes, we filmed their motion at framerates up to 250,000 fps. We never observed vibration of the vocal folds, only of the vocal membranes. Simple threshold detection did not reliably extract the moving tips of the transparent vocal membranes that passed over the underlying vocal fold; therefore, we trained a neural network for posture analysis (DeepLabCut, see Materials and methods) that reliably detected the vocal membrane edge along the glottal opening in millions of frames (S2 Movie). The *f*_o_ of these oscillations increased linearly with the *f*_o_ of the produced sound (Fig 2F and S1 Table) at a range of 10 to 20 kHz.

An important characteristic of myoelastic-aerodynamic systems [29] is the minimal pressure, aka phonation threshold pressure (PTP), needed to induce a behavior state change of the dynamical system from steady state to oscillating limit cycle. A lower PTP means a more efficient energy conversion from air flow to acoustic pressure [30]. To accurately measure the PTP in excised larynx or syrinx experiments, a slow increase of bronchial pressure is typically applied [31]. Indeed, we could determine the PTP of the ventricular folds during slow 1 kPa/s increases to be 3.99 ± 0.87 kPa (*N* = 4).

**Fig 3:**
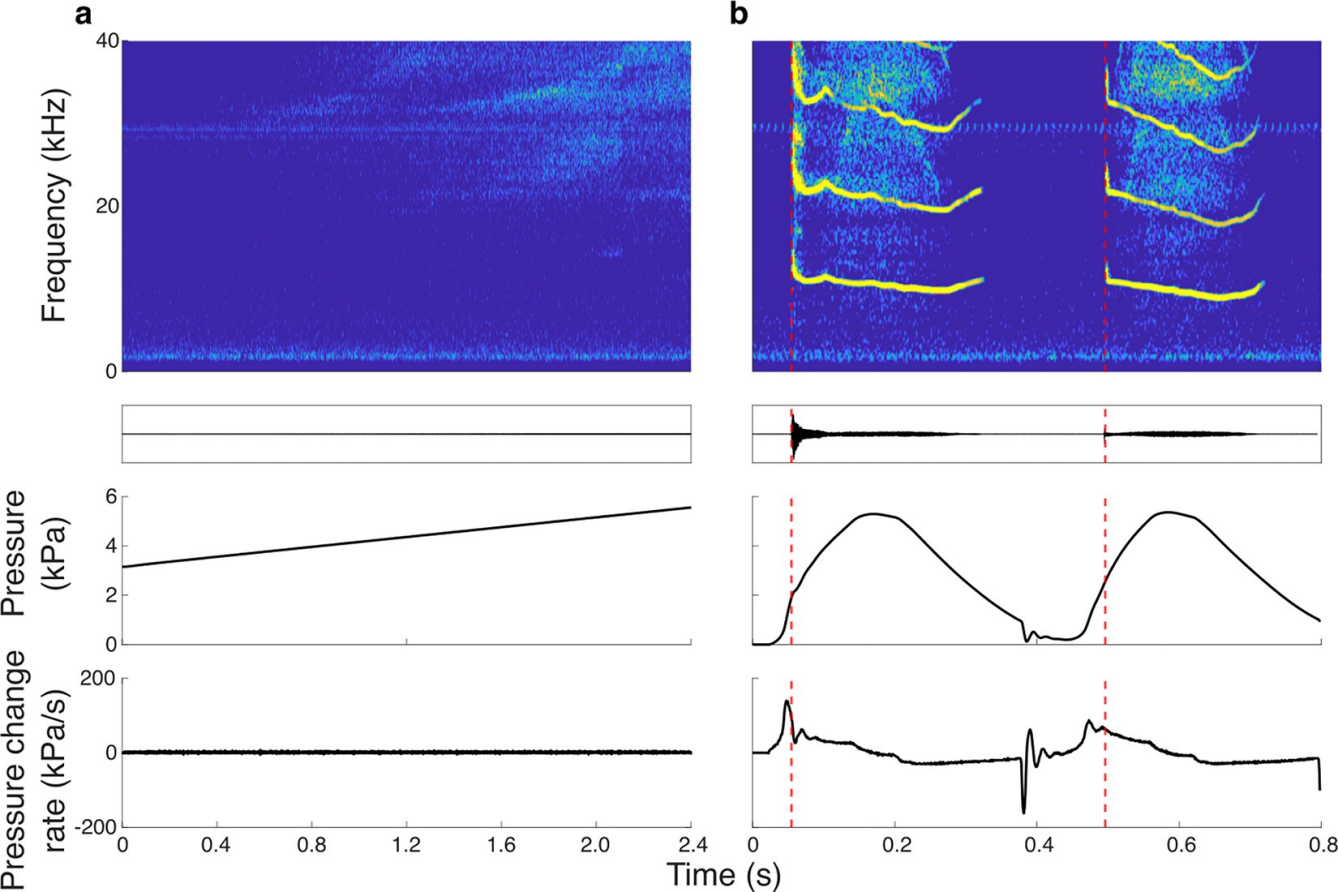
Oscillation onset of vocal membranes requires fast pressure modulation compared to ventricular folds. (a) Sound spectrograms during slow 1 kPa/s bronchial pressure show that vocal membranes did not oscillate during slow ramps (in 3 out of 4 individuals). (b) Driven by fast pressure modulation, vocal membranes reliably vibrated. Red vertical dashed lines show the onset for PTP detection. The data underlying a and b can be found in S4 and S5 Data files.

However, for vocal membrane vibration, the onset requirements were strikingly different. Interestingly, we could not consistently induce self-sustained oscillation of the vocal membranes at slow pressure ramps (Fig 3A). Only when we drove the larynges with faster pressure patterns that more closely resembled in vivo pressure pulses measured in *E*. *fuscus* [14] did we succeed in consistent induction of vocal membrane oscillation and thereby sound (Fig 3B). Of the 5 specimens, we applied both slow and fast pressure ramps, all 5 showed vocal membrane oscillation onset during fast pressure ramps, but only 1 during slow. The PTP was 3.23 ± 1.41 kPa (*N* = 5) at a pressure rate change of 130.8 ± 85.0 kPa/s. Thus, for bat vocal membranes, two pressure requirements need to be met for the system to bifurcate to stable limit cycle oscillation: a minimal pressure and a high pressure rate of change.

**Fig 4:**
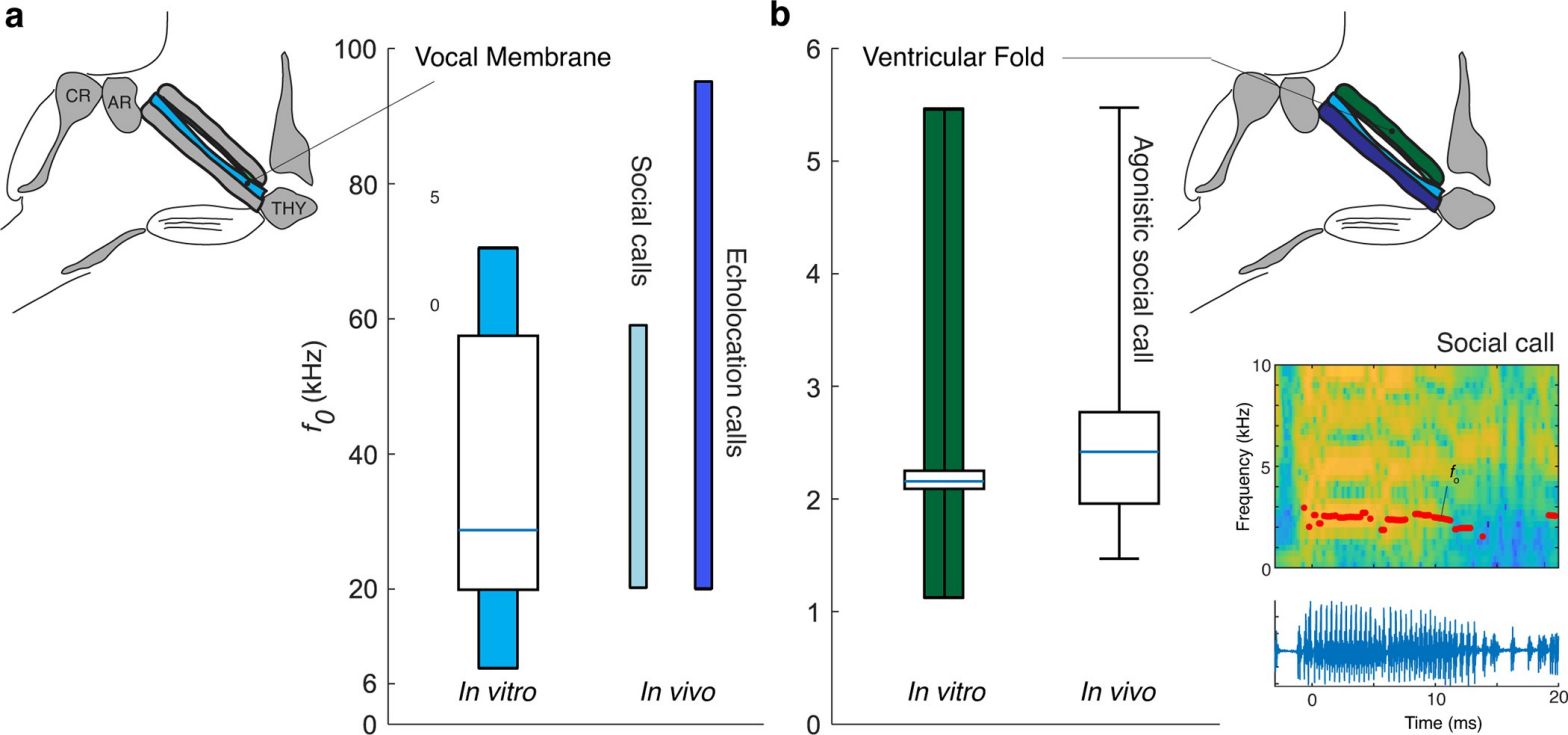
The vocal range of laryngeal structures in vitro corresponds to frequency ranges of distinct social calls in Daubenton’s bat. (a) Vocal membrane *f*_*o*_ range in vitro (blue vertical bar) compared to reported in vivo range for social [8] and echolocation calls [24] of *M*. *daubentonii*. (b) Ventricular fold *f*_*o*_ range in vitro (green vertical bar) corresponds well to *f*_*o*_ range of agonistic social calls of *M*. *daubentonii*. Boxplot whiskers indicate range. For values see S2 Table. Inset shows a spectrogram and oscillogram of an agonistic social call. Abbreviations as in Fig 1. The data underlying a and b can be found in S6 Data.

At first approximation, the distinct *f*_o_ ranges for vocal membranes (10 to 20 kHz) and ventricular fold (1 to 5 kHz) produced by the different structures in the larynx in vitro correspond to the *f*_o_ ranges of the distinct call types used by most Vespertilionids; echolocation versus social communication calls. However, echolocation calls in *M*. *daubentonii* can extend much higher with *f*_o_’s up to 95 kHz [24]. Fundamental frequency control in bats, and mammals in general, is mostly achieved by contracting the CT muscle (Fig 1B) [2,11,14]. We mimicked contraction of the CT muscle by rotating the cricoid cartilage caudal (See Materials and methods), thereby lengthening an increasing tension in both vocal folds and vocal membranes (Fig 1B). Indeed, this rotation led to an upward extension of the *f*_o_ range to 70 kHz. Thus, in vitro the vocal membranes oscillated from 10 to 70 kHz, which overlaps well with the *f*_*o*_ range in vivo of both echolocation and several types of social calls of *M*. *daubentonii* (Fig 4A).

Next, we recorded low-frequency agonistic calls of 3 individuals of *M*. *daubentonii*. These very short (<2 ms) calls are often described as broadband, noisy sounds [8], but they have a harmonic structure of which the *f*_o_ distribution was 1 to 5 kHz (Fig 4B, see Materials and methods). Thus, the *f*_*o*_ range of in vivo agonistic social calls overlaps with the in vitro vibration of the ventricular folds (Fig 4B), which strongly suggests that these structures are responsible for the generation of low-frequency agonistic calls.

## Discussion

By filming the bat larynx in vitro with ultra-high-speed video up to 250,000 fps and using deep learning networks to extract vocal membrane motion, we provide the first direct observations that vocal membranes exhibit flow-induced self-sustained vibrations to produce echolocation calls in Daubenton’s bats. Furthermore, we show that both vocal membrane and ventricular folds vibrate to produce sound and at distinctly different frequency ranges. The vocal membranes generate 10 to 70 kHz high frequencies in the echolocation and social call range, while the ventricular folds produce 1 to 5 kHz low-frequencies in the range of agonistic social calls.

Mammalian vocal membranes have been hypothesized to serve 3 specific purposes [18] that we can now test experimentally on bats. Firstly, vocal membranes supposedly increase *f*_o_ by uncoupling the vocal membrane vibration from vocal fold vibration. Our data confirm high-frequency vocal membrane vibration and the in vitro range *f*_o_ without CT modulation (10 to 20 kHz) correspond well to the in vivo range of 8 to 20 kHz after bilateral ablation of the superior laryngeal nerve in *E*. *fuscus* [14]. In contrast to vocal membranes in marmosets [19], we observed that bat vocal membranes vibrated completely uncoupled from the vocal folds and did not observe any vocal fold motion at all. Second, vocal membranes can supposedly reduce the PTP and thereby increase vocal efficiency. Our experimental data contradicts these model-based suggestions. The vocal membranes had a PTP of 3.22 ± 1.41 kPa in vitro, which compares well to PTP in vivo 2.5 to 4.0 kPa in *E*. *fuscus* [14]. This species is twice the weight of *M*. *daubentonii* and thus its PTP may deviate from *M*. *daubentonii*. However, when comparing across mammals, such PTP values are, if anything, on the high side and certainly not lower. The unsteady aerodynamic conditions required to initiate vocal membrane vibration are fascinating. Low Reynolds number airfoils show peaks in drag and lift coefficients due to rapid acceleration of relative airspeed [32], which are preceded by the maximum acceleration points [33] in a manner that mirrors the pressure speed profiles preceding the vocal membrane vibration onset in this study. Although the flow conditions are different and our observations are preliminary, they emphasize the need for further investigation of the role of unsteady aerodynamics effects in bat vocalizations. Thirdly, the vocal membranes supposedly support the production of broadband chaotic signals via increased oscillatory coupling [18]. Our data does not support this hypothesis in bats either. We did not observe mechanical coupling between vocal folds and vocal membranes, and although we did not quantify this specifically, we did not observe deterministic chaotic signals.

The role of the peculiar ventricular apical membranes remains unclear. The ventricular and vocal membranes form a drumhead with a narrow slit over the ventricle of Morgagni [15], this configuration opens to the hypothesis that the ventricle of Morgagni acts as a cavity that generated a shallow cavity whistle [26] for echolocation calls. However, our data clearly shows that removing the ventricular folds and membranes—and thereby the ventricle—results in high-frequency sounds by vocal membrane oscillation. Therefore, they were not essential for sound production, but this does not exclude that they play a role. Perhaps, the ventricular membranes are coupled to vocal membrane oscillation during echolocation calls. Unfortunately, we could not directly observe the ventricular membranes in our experiments as they were either obscured by the ventricular folds and removed—together with the ventricular folds—when observing the vocal membranes and folds. Direct observations in vitro could involve a hemilarynx experiment, where the larynx is halved and closed by a glass plate through which the oscillations can be observed [34].

Anatomical adaptations in the bat larynx, such as the ossified cricoid and thyroid in combination with hypertrophied muscles, are purportedly adaptations to high pressures in the larynx during sound production [25]. However, an acoustic pressure of maximally 200 Pa (= 140 dB re. 20 μPa) [35] and maximal 8 kPa bronchial air pressures [14] do not exert much stress on bony structures with tensile strengths in the MPa range [36]. Instead, we propose that the ossification results from a strong selection on these structures to reduce weight while maintaining structural strength. The superfast CT muscles can power the rapid motion needed during feeding buzzes, but their speed trades off with force [2,37,38]. As a result, superfast muscles are exceptionally weak [37] and produce over 50 times lower tetanic stresses compared to normal skeletal muscles [39]. Muscular hypertrophy can partially compensate for the low area-specific force of superfast vocal muscles in bats [2] as it increases the cross-section area and thus the total force.

Taken together, we propose the evolutionary scenario that many laryngeal morphological adaptations in echolocating bats are the result of selection for producing (1) high-frequency and (2) rapid echolocation calls to catch fast moving prey. This scenario would be concurrently followed by a complimentary specialization of the auditory system that affords bats sensitive hearing at high frequencies and over a wide frequency range [40]. First, a strong selection to increase spatial resolution [3] led to an increase in *f*_*o*_ by reducing the mass of the vibrating vocal membranes. Second, a strong selection to increase call repetition rate led to very low muscle force [2]. The reduced force was compensated by higher cross-sectional area (CSA), i.e., a hypertrophied muscle, and the actuated mass was reduced to require less force: The vocal folds reduced in mass and both thyroid and cricoid reduced in size and became ossified to withstand large bending moments during acceleration. Lastly, the reduced thyroid was replaced by the cricothyroid membrane to have a flexible, airtight trachea. Taken together, these adaptations allowed the production of ultrasonic calls with fast FM that could be repeated above 200 Hz for catching erratic airborne prey in the dark.

The vocal membranes achieve unparalleled high voiced *f*_*o*_ in bats. However, the vocal range of vocal membrane produced echolocation calls with 10 to 95 kHz in Daubenton’s bat is only 3 to 4 octaves and thereby comparable to other mammals [10]. When considering only vocal membrane produced sounds, we expect the vocal range for all bats to fit within 3 to 4 octaves. As a consequence, we do not expect the material properties of the vocal membranes to be significantly different. However, because smaller strains in muscles allow faster motion, an increased stiffness would require a smaller range of motion to achieve the same vocal range [10]. Therefore, a stiffer vocal membrane would allow faster FM and call repetition rates at the same frequency bandwidth, but this remains to be tested.

There is only limited known ways to lower *f*_*o*_ for mammals. First, vocal folds can exhibit different vibratory patterns, aka registers, due to differential posturing by laryngeal muscles [22,23]. In humans, the lowest register is the vocal fry register. The excised horseshoe bat larynx produced distinctly different frequencies that were suggested to be different registers [11], but no laryngeal dynamics were measured to confirm this. In contrast, our data suggest that in FM bats, echolocation calls and agonistic social calls are not caused by different vocal membranes registers, but by using different laryngeal structures. The mechanism by which ventricular folds decrease *f*_*o*_ in other mammals is by coupled oscillation to vocal folds, as in tigers [41], grunting pigs [21], human throat singing [42], and metal growling [43]. In our preparation, we did not see vocal fold vibration in any condition and were not able to observe vocal folds during ventricular fold oscillation. As such, we cannot be conclusive that the lower *f*_*o*_ is the result of mechanical coupling between laryngeal structures. However, because we could not get the vocal folds to oscillate, we venture to speculate that in bats, the ventricular folds have taken on the role of lower frequency vibrations.

An additional effect of high *f*_*o*_ is highly directional sound emission, i.e., sound pressure attenuates rapidly at angles away from the main broadcast axis. This has substantial benefits for navigation through echolocation [44], but likely becomes disadvantageous for social communication as the sender generally wishes to broadcast as broadly as possible depending on the context [45]. Thus, there likely is a strong opposing evolutionary drive for echolocation calls versus social calls. Echolocation favors high frequencies for spatial resolution and high directionality, while communication favors low frequencies for low directionality and low atmospheric attenuation. This duality may then have facilitated the evolution of separate vocal sub-structures with distinctly different sound producing purposes in bats. Likewise, fruit bats of the genus *Rousettus* echolocate by tongue clicks and communicate via laryngeal sounds [46], indicating a similar duality between echolocation and social call production. Together, the different mechanisms vastly expand the vocal range in bats and provide a rich substrate for vocal communication.

## Materials and methods

### Subjects

We used the larynges of 8 adult specimens of *M*. *daubentonii* in total (6 males, 2 females). Animals were caught under license 2020–9239 from the Ministry of Environment. Animals were housed in bat keeping facilities at 11L:13D photoperiod at approximately 22°C and 60% relative humidity. All experiments were conducted at the University of Southern Denmark and were in accordance with the Danish Animal Experiments Inspectorate (Copenhagen, Denmark).

### Larynx dissection and preparation

All animals were euthanized with isoflurane (Baxter laboratories). The trachea, larynx, and surrounding tissue were dissected in ice-cold oxygenated buffer (150 mM NaCl, 2.5 mM KCl, 4 mM CaCl2, 1 mM NaH2PO4, 1 mM MgSO4, 10 mM HEPES, 12 mM Glucose, pH 7.4 adjusted with a 1 M Trizma solution). Five specimens (MD10, MD11, MD21, MD22, and MD23) were flash-frozen in liquid nitrogen and stored at −80°C. Two specimens (MD13 and MD14) were used fresh in the setup described below. For 1 specimen (MD12), the larynx was transferred to a sylgard-covered petri dish on ice for inspection under a stereomicroscope (M165-FC, Leica Microsystems). This specimen was then also flash-frozen in liquid nitrogen and stored at −80°C. Later, this specimen was thawed and fixed in 4% PFA on a roller for cross-sections.

Before an experiment, we thawed the tissue in a refrigerator and then submerged it in refrigerated ringer’s solution in a dish on ice and removed additional tissue surrounding the larynx and trachea. We then mounted the larynx on a rounded, blunted 21G needle (Sterican, 0.8 × 40 mm). The larynx was slid over the blunt needle until the caudal edge of the cricoid touched the tube exit and secured with a 10 to 0 monofilament suture (AroSurgical Instruments, California, United States of America) around the trachea.

### Experimental setup

We mounted the larynges in the excised larynx setup described previously [26,27]. The setup allows for running humidified air through the larynx at precisely controlled pressures (model PCD, Alicat Scientific) while controlling the configuration of the larynx with micromanipulators and recording any sound produced. For recording the sound, we used a 1/4-inch pressure microphone-preamplifier assembly (model 46BD, frequency response ± 1 dB 10 Hz to 25 kHz and ± 2 dB 4 Hz to 70 kHz, G.R.A.S., Denmark). The positions of the larynx and microphone were fixed relative to each other during an experiment and placed horizontally at 22 to 44 mm from the larynx. The microphone signal was amplified (12AQ, G.R.A.S., Denmark) and calibrated before each experiment (Calibrator 42AB, G.R.A.S., Denmark). The sound, pressure, and flow signals were low pass filtered at 100, 10, and 10 kHz, respectively (filter model EF502 low pass filter DC– 100 kHz and EF120 low pass filter DC– 10 kHz, Thorlabs, USA), and digitized at 250 kHz (USB 6259, 16 bit, National Instruments, Austin, Texas, USA).

To capture the laryngeal configuration during the experiments, we used a Leica DC425 camera mounted on the stereomicroscope, controlled using LAS (Leica Application Suite Version 4.7.0, Leica Microsystems, Switzerland). To record tissue vibration, we used a high-speed camera (FASTCAM SA1.1, Photron, Tokyo, Japan) filming at 10,000 to 20,000 fps for ventricular folds and 100,000 to 250,000 fps for vocal membranes, controlled by Photron FASTCAM Viewer 4. For illumination, we used a Leica GLS150 lamp through a liquid light guide connected to the stereomicroscope (static images) or a Thorlabs plasma light source (HPLS200 Series) (high-speed-imaging). All control and analysis software were written in MATLAB (MathWorks).

### Excised larynx phonation protocol

We removed the epiglottis to give an unobstructed view of the ventricular folds and make adduction of the arytenoids easier. To induce ventricular fold vibration, we applied a linear increase in bronchial pressure from 0 to 6 kPa at a speed of 1 kPa/s. We wanted to minimize the amount of air flowing over the delicate laryngeal structures to prevent them from drying out. Because the PTP values were rather high, we did not always start at 0 kPa, but sometimes at 3 kPa. Ventricular fold vibration was induced in 4 larynges (MD10, MD11, MD13, and MD23). We then turned on the plasma light source and repeated this ramp while triggering the camera when the pressure was passing the PTP. In 3 of these (MD11, MD13, and MD23), we successfully filmed their vibration.

To expose the vocal membranes, we carefully cut in a horizontal plane between the ventricular and vocal membranes with adventitia scissors (S&T surgical instruments, Switzerland) through the ventricle of Morgagni. To induce their vibration, we applied a slow pressure ramp from 0 to 7 kPa at 1 kPa/s. This type of pressure function only yielded oscillation for 1 out of the first 4 individuals, and we did not apply it for the last 2 to minimize experimental time. Next, we applied a sequence of 4, 300 ms duration fast pressure modulation between 0 and 4 kPa. This readily resulted in oscillation in 5 specimens (MD10, MD11, MD13, MD14, and MD23). Because we needed to film at rates up to 250,000 fps, we only had short buffer available and sometimes needed several runs to trigger the camera during vocal membrane vibration with correct lighting conditions. We successfully filmed vocal membrane oscillation in 4 animals.

To increase the *f*_*o*_ of the vocal membrane vibrations, we mimicked cricothyroid muscle contraction. We applied 5 to 7 kPa pressure for 1.5 seconds and manually rotated the thyroid downward to increase the tension of the vocal fold and membrane in 5 individuals (MD11, MD13, MD14, MD22, and MD23). Since the yin algorithm tends to fail for *f*_*o*_’s above 1 quarter of the sampling rate [47], we instead extracted them using the time frequency ridge detection function in MATLAB (tfridge) on spectrograms of the sound signal (nfft = 2,048, overlap = 50%, Hamming window) [26].

### Glottovibrogram construction

Each video was rotated to make the glottal midline vertical and cropped around the glottis. We then calculated the opening of the vocal folds as a function of anterior-posterior position (AP) and time, i.e., the glottovibrogram (GVG), by automated detection of the glottis shape per image. For the ventricular folds, the glottis was defined as all pixels below a manually set threshold gray value. The resulting logical image was horizontally and vertically dilated with a 2-pixel line (*imdilate* function in MATLAB) and filled (*imfill*), which resulted in an outline of the glottis. The glottis width was the sum of the vertical opening pixels scaled for magnification. To determine the position of the vocal membrane edges, we could not use a simple image grayscale threshold, because the vocal membranes were too translucent, and the trailing edge was crossing the underlying vocal folds with nearly the same pixel values. This led to erroneous detection of the thin vocal membrane parts as glottis. Instead, we trained a deep learning model to detect the vocal membrane edges using the deep learning Python package DeepLabCut (2.2b) [48,49]. We digitally superimposed 8 to 10 equidistantly spaced dashed horizontal lines on the videos and trained the network on detecting where the vocal membrane edges crossed these lines. The superimposed lines were used to fix the detections vertically as we were only interested in the horizontal movement of the vocal membranes. After training for 1 million iterations, the videos were analyzed, resulting in pixel coordinates for points along the glottal edge for each analyzed frame.

To calculate the *f*_*o*_, we first determined the anterior-posterior (AP) location where the mean opening was maximal. Then, we extracted the opening at this location along the AP axis from the GVG. We resampled all other physiological signals (pressure, sound) to the framerate of the video (*resample* function in MATLAB). The *f*_*o*_ of the sound and glottal opening signal was determined using the yin algorithm [47], combining signal power and aperiodicity criteria to extract *f*_*o*_ per 10 frames.

### Signal analysis

To determine PTP and S_ptp_, we first low pass filtered the pressure signal at 500 Hz with a sixth order Butterworth filter (*butter* and *filtfilt* functions in MATLAB) to eradicate any high-frequency fluctuations. The rate or speed of the pressure change was then calculated by first finding the pressure change between time steps (*diff* function in MATLAB), this value was then multiplied by the acquisition rate (250 kHz) to get the pressure speed (per second rate of pressure change). We defined PTP and S_ptp_ as the pressure and pressure speed at the time where the sound power crossed 0.2 mPa.

### In vivo social call recordings

Because we could not find detailed quantification of the low-frequency calls of *M*. *daubentonii* in the literature, we recorded 9 additional males in Odense, Denmark caught under license 2021–1194. Daubenton’s bats do not spontaneously produce low-frequency calls as easily as, e.g., *Pipistrellus pygmaeus*, and only 3 individuals produced such calls when (1) they were joined with others into 1 enclosure after daily weighting; or (2) when stroked roosting in the large flight cage at SDU. We recorded calls with an Olympus LS-100 24-bit recorder at sampling rate of 96 kHz and a Grass 40BF ¼” microphone connected to a Avisoft 16-bit USG at 375 kHz. We selected small segments that included calls and extracted the *f*_*o*_ of the sound with the yin algorithm [47].

### Statistics

All values listed are mean ± SD. The correlation between the *f*_*o*_ of sound and vocal fold vibrations was established with linear regression (*regress* function) in MATLAB (MathWorks). The boxplots were constructed using the MATLAB toolbox IoSR (v.2.8, Institute of Sound Recording, University of Surrey, 2016), with no limit for outliers, meaning horizontal lines indicate minimum, maximum, median, and interquartile range.

## Supporting information

S1 Table Descriptive statistics of *f*_*o*_ regressions vocal membrane vibration versus sound in Fig 2F. (DOCX)

S2 Table Sound *f*_*o*_ ranges for different call types and laryngeal performance in vitro in *Myotis daubentonii*. (DOCX)

S3 Table Phonation threshold pressures (PTP) and pressure speed at PTP (S_ptp_) in vitro. (DOCX)

S1 Movie Ventricular fold oscillation during sound production. Individual MD13; filmed at 20,000 frames per second.
(MP4)

S2 Movie Vocal membrane oscillation during sound production. The edge of the vocal membranes are detected at 10 locations with a neural network. Individual MD13; filmed at 125,000 frames per second.
(MP4)

S1 Data The data underlying Fig 2C, glottovibrogram of ventricular fold vibration. File contains data points for time and AP position axis, as well as the width of the opening in pixels and millimeters at the corresponding AP positions.
(MAT)

S2 Data The data underlying Fig 2E, Glottovibrogram of vocal membrane vibration. File contains data points for time and AP position axis, as well as the width of the opening in pixels and millimeters at the corresponding AP positions.
(MAT)

S3 Data The data underlying Fig 2F, *f*_*o*_ of vibration and resulting sound for ventricular folds and vocal membranes. File contains data points for *f*_*o*_ of vibration (x) and sound (y).
(MAT)

S4 Data The data underlying Fig 3A, sound spectrogram during slow 1 KPa/s bronchial pressure ramp. File contains data points for the spectrogram frequency, power, and time.
(MAT)

S5 Data The data underlying Fig 3B, sound spectrogram during fast bronchial pressure modulation. File contains data points for the spectrogram frequency, power, and time, as well as the onset times. Stored as MATLAB data file (.mat).
(MAT)

S6 Data The data underlying Fig 4A and 4B, vocal membrane *f*_*o*_ range in vitro, ventricular fold *f*_*o*_ range in vitro, and *f*_*o*_ range of agonistic social calls of *M*. *daubentonii*. File contains extracted *f*_*o*_’s.
(MAT)

## Abbreviations

AP: anterior-posterior position
CSA: cross-sectional area
GVG: glottovibrogram
PTP: phonation threshold pressure

## References

[1] Ratcliffe JM, Elemans CPH, Jakobsen L, Surlykke A. How the bat got its buzz. Biol Lett. 2013;9(2). ARTN 20121031. doi: 10.1098/rsbl.2012.1031 WOS:000313958000002. 23302868 PMC3639754

[2] Elemans CP, Mead AF, Jakobsen L, Ratcliffe JM. Superfast muscles set maximum call rate in echolocating bats. Science. 2011;333(6051):1885–8. Epub 2011/10/01. doi: 10.1126/science.1207309 . 21960635

[3] Schnitzler H-U, Kalko EKV. Echolocation by Insect-Eating Bats. Bioscience. 2001;51:7. doi: 10.1641/0006-3568(2001)051[0557:Ebieb]2.0.Co;2

[4] Siemers BM, Schnitzler HU. Natterer’s Bat (Myotis nattereri Kuhl, 1818) Hawks for Prey Close to Vegetation Using Echolocation Signals of Very Broad Bandwidth. Behav Ecol Sociobiol. 2000;47(6):400–412.

[5] Jones G, Holderied MW. Bat echolocation calls: adaptation and convergent evolution. Proc Biol Sci. 2007;274(1612):905–12. Epub 2007/01/26. doi: 10.1098/rspb.2006.0200 ; PubMed Central PMCID: PMC1919403. 17251105 PMC1919403

[6] Schmieder DA, Kingston T, Hashim R, Siemers BM. Breaking the trade-off: rainforest bats maximize bandwidth and repetition rate of echolocation calls as they approach prey. Biol Lett. 2010;6(5):604–9. Epub 2010/04/02. doi: 10.1098/rsbl.2010.0114 ; PubMed Central PMCID: PMC2936139. 20356884 PMC2936139

[7] Smotherman M, Knornschild M, Smarsh G, Bohn K. The origins and diversity of bat songs. J Comp Physiol A Neuroethol Sens Neural Behav Physiol. 2016;202(8):535–54. Epub 2016/06/29. doi: 10.1007/s00359-016-1105-0 . 27350360

[8] Pfalzer G, Kusch J. Structure and variability of bat social calls: implications for specificity and individual recognition. J Zool. 2003;261:21–33. doi: 10.1017/S0952836903003935 WOS:000185684700004.

[9] Barclay RMR. Social Behavior of the Little Brown Bat, Myotis lucifugus: II Vocal Communication. Behav Ecol Sociobiol. 1979;6(2):137–146.

[10] Titze I, Riede T, Mau T. Predicting Achievable Fundamental Frequency Ranges in Vocalization Across Species. PLoS Comput Biol. 2016;12(6):e1004907. Epub 2016/06/17. doi: 10.1371/journal.pcbi.1004907 ; PubMed Central PMCID: PMC4911068. 27309543 PMC4911068

[11] Kobayasi KI, Hage SR, Berquist S, Feng J, Zhang S, Metzner W. Behavioural and neurobiological implications of linear and non-linear features in larynx phonations of horseshoe bats. Nat Commun. 2012;3:1184. Epub 2012/11/15. doi: 10.1038/ncomms2165 ; PubMed Central PMCID: PMC3552533. 23149729 PMC3552533

[12] Metzner W, Schuller G. Vocal control in echolocating bats. Handbook of Mammalian Vocalization—An Integrative Neuroscience Approach. Handbook of Behavioral Neuroscience. 2010:403–415.

[13] Griffiths TA. Modification of M. cricothyroideus and the Larynx in the Mormoopidae, with Reference to Amplification of High-Frequency Pulses. Journal of Mammalogy. 1978;59: 724–730. doi: 10.2307/1380137

[14] Suthers RA, Fattu JM. Mechanisms of sound productino echolocating bats. Am Zool. 1973;13:1215–1226.

[15] Novick A, Griffin DR. Laryngeal mechanisms in bats for the production of orientation sounds. J Exp Zool. 1961;148:125–45. Epub 1961/11/01. doi: 10.1002/jez.1401480203 . 14480574

[16] Elias H. Zur anatomie des Kehlkopfes der Mikrochiropteren. Morphol Jahrb. 1907;37:70–119.

[17] Griffiths TA. Compative laryngeal anatomy. Mammalia. 1983;47:3.

[18] Mergell P, Fitch WT, Herzel H. Modeling the role of nonhuman vocal membranes in phonation. J Acoust Soc Am. 1999;105(3):2020–8. Epub 1999/03/25. doi: 10.1121/1.426735 . 10089619

[19] Zhang YS, Takahashi DY, Liao DA, Ghazanfar AA, Elemans CPH. Vocal state change through laryngeal development. Nat Commun. 2019;10(1):4592. Epub 2019/10/11. doi: 10.1038/s41467-019-12588-6 ; PubMed Central PMCID: PMC6785551. 31597928 PMC6785551

[20] Bailly L, Henrich N, Pelorson X. Vocal fold and ventricular fold vibration in period-doubling phonation: physiological description and aerodynamic modeling. J Acoust Soc Am. 2010;127(5):3212–22. Epub 2010/12/02. doi: 10.1121/1.3365220 . 21117769

[21] Herbst CT, Nishimura T, Garcia M, Migimatsu K, Tokuda IT. Effect of Ventricular Folds on Vocalization Fundamental Frequency in Domestic Pigs (Sus scrofa domesticus). J Voice. 2021;35(5):805 e1–e15. Epub 2021/01/04. doi: 10.1016/j.jvoice.2020.01.013 . 33388229

[22] Roubeau B, Henrich N, Castellengo M. Laryngeal vibratory mechanisms: the notion of vocal register revisited. J Voice. 2009;23(4):425–38. Epub 2008/06/10. doi: 10.1016/j.jvoice.2007.10.014 . 18538982

[23] Herbst CT. Registers—The Snake Pit of Voice Pedagogy. Part 1: Proprioception, Perception, And Laryngeal Mechanisms. J Sing. 2020;77: 175–190.

[24] Kalko EKV, Schnitzler HU. The Echolocation and Hunting Behavior of Daubenton Bat, Myotis daubentoni. Behav Ecol Sociobiol. 1989;24(4):225–238. doi: 10.1007/Bf00295202 WOS:A1989U087600004.

[25] Carter RT. Reinforcement of the larynx and trachea in echolocating and non-echolocating bats. J Anat. 2020;237(3):495–503. Epub 2020/04/23. doi: 10.1111/joa.13204 ; PubMed Central PMCID: PMC7476186. 32319086 PMC7476186

[26] Håkansson J, Jiang W, Xue Q, Zheng X, Ding M, Agarwal AA, et al. Aerodynamics and motor control of ultrasonic vocalizations for social communication in mice and rats. BMC Biol. 2022;20(1):3. Epub 2022/01/09. doi: 10.1186/s12915-021-01185-z ; PubMed Central PMCID: PMC8742360. 34996429 PMC8742360

[27] Mahrt E, Agarwal A, Perkel D, Portfors C, Elemans CP. Mice produce ultrasonic vocalizations by intra-laryngeal planar impinging jets. Curr Biol. 2016;26(19):R880–R1. Epub 2016/10/12. doi: 10.1016/j.cub.2016.08.032 . 27728788

[28] Elemans CP, Rasmussen JH, Herbst CT, During DN, Zollinger SA, Brumm H, et al. Universal mechanisms of sound production and control in birds and mammals. Nat Commun. 2015;6:8978. Epub 2015/11/28. doi: 10.1038/ncomms9978 ; PubMed Central PMCID: PMC4674827. 26612008 PMC4674827

[29] Svec JG, Schutte HK, Chen CJ, Titze IR. Integrative Insights into the Myoelastic-Aerodynamic Theory and Acoustics of Phonation. Scientific Tribute to Donald G. Miller. J Voice. 2021. Epub 2021/03/22. doi: 10.1016/j.jvoice.2021.01.023 . 33744068

[30] Titze IR. The Physics of Small-Amplitude Oscillation of the Vocal Folds. J Acoust Soc Am. 1988;83(4):1536–1552. doi: 10.1121/1.395910 WOS:A1988M944300043. 3372869

[31] Mau T, Muhlestein J, Callahan S, Weinheimer KT, Chan RW. Phonation threshold pressure and flow in excised human larynges. Laryngoscope. 2011;121(8):1743–51. Epub 2011/07/28. doi: 10.1002/lary.21880 ; PubMed Central PMCID: PMC3146025. 21792964 PMC3146025

[32] Dickinson MH, Gotz KG. Unsteady Aerodynamic Performance of Model Wings at Low Reynolds-Numbers. J Exp Biol. 1993;174:45–64. WOS:A1993KP15700003. 10.1242/jeb.192.1.179 9317589

[33] Hamdani H, Sun M. Aerodynamic forces and flow structures of an airfoil in some unsteady motions at small Reynolds number. Acta Mech. 2000;145:173–187.

[34] Alipour F, Scherer RC. Dynamic glottal pressures in an excised hemilarynx model. J Voice. 2000;14(4):443–454. doi: 10.1016/s0892-1997(00)80002-8 . 11130103

[35] Jakobsen L, Christensen-Dalsgaard J, Juhl PM, Elemans CPH. How Loud Can you go? Physical and Physiological Constraints to Producing High Sound Pressures in Animal Vocalizations. Front Ecol Evol. 2021:9. doi: 10.3389/fevo.2021.657254

[36] Hart NH, Nimphius S, Rantalainen T, Ireland A, Siafarikas A, Newton RU. Mechanical basis of bone strength: influence of bone material, bone structure and muscle action. J Musculoskel Neuron. 2017;17(3):114–39. WOS:000410545600001. 28860414 PMC5601257

[37] Mead AF, Osinalde N, Ortenblad N, Nielsen J, Brewer J, Vellema M, et al. Fundamental constraints in synchronous muscle limit superfast motor control in vertebrates. eLife. 2017;6. Epub 2017/11/23. doi: 10.7554/eLife.29425 ; PubMed Central PMCID: PMC5699865. 29165242 PMC5699865

[38] Rome LC, Cook C, Syme DA, Connaughton MA, Ashley-Ross M, Klimov A, et al. Trading force for speed: Why superfast crossbridge kinetics leads to superlow forces. Proc Natl Acad Sci U S A. 1999;96(10):5826–5831. doi: 10.1073/pnas.96.10.5826 WOS:000080246500091. 10318969 PMC21945

[39] Adam I, Maxwell A, Rossler H, Hansen EB, Vellema M, Brewer J, et al. One-to-one innervation of vocal muscles allows precise control of birdsong. Curr Biol. 2021;31(14):3115–24 e5. Epub 2021/06/06. doi: 10.1016/j.cub.2021.05.008 ; PubMed Central PMCID: PMC8319070. 34089645 PMC8319070

[40] Fay J. Hearing by bats. Popper AN, editor. New York: Springen-Verlag; 1995.

[41] Titze IR, Fitch WT, Hunter EJ, Alipour F, Montequin D, Armstrong DL, et al. Vocal power and pressure-flow relationships in excised tiger larynges. J Exp Biol. 2010;213(Pt 22):3866–73. Epub 2010/11/03. doi: 10.1242/jeb.044982 ; PubMed Central PMCID: PMC2966350. 21037066 PMC2966350

[42] Lindestad P-Å, Södersten M, Merker B, Granqvist S. Voice Source Characteristics in Mongolian “Throat Singing” Studied with High-Speed Imaging Technique, Acoustic Spectra, and Inverse Filtering. J Voice. 2001;15(1):78–85. doi: 10.1016/S0892-1997(01)00008-X 12269637

[43] Eckers C, Hütz D, Kob M, Murphy P, Houben D, Lehnert B. Voice production in death metal singers. Nag/Daga. 2009:1747–1750.

[44] Jakobsen L, Ratcliffe JM, Surlykke A. Convergent acoustic field of view in echolocating bats. Nature. 2013;493(7430):93–96. doi: 10.1038/nature11664 WOS:000312933800037. 23172147

[45] Patricelli GL, Dantzker MS, Bradbury JW. Differences in acoustic directionality among vocalizations of the male red-winged blackbird (Agelaius pheoniceus) are related to function in communication. Behav Ecol Sociobiol. 2007;61(7):1099–1110. doi: 10.1007/s00265-006-0343-5

[46] Lee WJ, Falk B, Chiu C, Krishnan A, Arbour JH, Moss CF. Tongue-driven sonar beam steering by a lingual-echolocating fruit bat. PLoS Biol. 2017;15(12):e2003148. Epub 2017/12/16. doi: 10.1371/journal.pbio.2003148 ; PubMed Central PMCID: PMC5774845. 29244805 PMC5774845

[47] de Cheveigne A, Kawahara H. YIN, a fundamental frequency estimator for speech and musica). J Acoust Soc Am. 2002;111:1917–1930. doi: 10.1121/1.1458024 12002874

[48] Mathis A, Mamidanna P, Cury KM, Abe T, Murthy VN, Mathis MW, et al. DeepLabCut: markerless pose estimation of user-defined body parts with deep learning. Nat Neurosci. 2018;21:1281–1289. doi: 10.1038/s41593-018-0209-y 30127430

[49] Nath T, Mathis A, Chen AC, Patel A, Bethge M, Mathis MW. Using DeepLabCut for 3D markerless pose estimation across species and behaviors. Nat Protoc. 2019;14:2152–2176. doi: 10.1038/s41596-019-0176-0 31227823

