# Supplementary material for: Bats expand their vocal range by recruiting different laryngeal structures for echolocation and social communication
Source: PLoS Biol. 2022 Nov 29;20(11):e3001881. doi: 10.1371/journal.pbio.3001881 (PMC9707786; doi:10.1371/journal.pbio.3001881)
Supplement: S1 Table — (DOCX) [file pbio.3001881.s001.docx]

**S1 Table. Descriptive statistics of *f_o_* regressions vocal membrane vibration versus sound in Fig 2f.**

|  |  | *f_o_,_sound_*=a **f_o_*_, vibration_+b | | Regression statistics | | |
| --- | --- | --- | --- | --- | --- | --- |
| **ID** | **Sex** | **a** | **b** | **R2** | **p** |  |
|  |  |  |  |  |  |  |
| MD10 | m | 0.923 | 1.301 | 0.922 | <<0.001 |  |
| MD11 | m | 0.995 | 0.054 | 0.991 | <<0.001 |  |
| MD13 | M | 0.855 | 2.857 | 0.895 | <<0.001 |  |
| MD23 | f | 1.002 | -0.022 | 0.995 | <<0.001 |  |
