# Supplementary material for: Bats expand their vocal range by recruiting different laryngeal structures for echolocation and social communication
Source: PLoS Biol. 2022 Nov 29;20(11):e3001881. doi: 10.1371/journal.pbio.3001881 (PMC9707786; doi:10.1371/journal.pbio.3001881)
Supplement: S2 Table — (DOCX) [file pbio.3001881.s002.docx]

**S2 Table. Sound *f_o_* ranges for different call types and laryngeal performance in vitro in *Myotis daubentonii*.**

| Context | Echolocation  [1] | Social  [2] | Vocal membrane in vitro | Agonistic social calls | Ventricular folds in vitro |
| --- | --- | --- | --- | --- | --- |
| Min *f_o_* | 20 | 20.2 | 8.28 | 1.4 | 1.13 |
| Max *f_o_* | 95 | 59.0 | 70.55 | 5.5 | 5.5 |

**References**

1. Pfalzer G, Kusch J. Structure and variability of bat social calls: implications for specificity and individual recognition. Journal of Zoology. 2003;261:21-33. doi: 10.1017/S0952836903003935. PubMed PMID: WOS:000185684700004.

2. Kalko EKV, Schnitzler HU. The Echolocation and Hunting Behavior of Daubenton Bat, Myotis-Daubentoni. Behavioral Ecology and Sociobiology. 1989;24(4):225-38. doi: Doi 10.1007/Bf00295202. PubMed PMID: WOS:A1989U087600004.
