# Supplementary material for: Bats expand their vocal range by recruiting different laryngeal structures for echolocation and social communication
Source: PLoS Biol. 2022 Nov 29;20(11):e3001881. doi: 10.1371/journal.pbio.3001881 (PMC9707786; doi:10.1371/journal.pbio.3001881)
Supplement: S3 Table — (DOCX) [file pbio.3001881.s003.docx]

**S3 Table. Phonation threshold pressures (PTP) and pressure speed at PTP (S_ptp_) in vitro.**

| ID | Vocal Membrane PTP (kPa) | Vocal Membrane s_ptp_  (kPa/s) | Ventricular folds  PTP  (kPa) |
| --- | --- | --- | --- |
| md10 | 1.91 | 76.0 | 5.40 |
| md11 | 2.83 | 58.0 | 3.20 |
| md13 | 3.17 | 295.3 | 3.33 |
| md14 | 5.93 | 109.1 | - |
| md23 | 2.33 | 115.4 | 4.03 |
| Mean ± S.D. | 3.24 ± 1.41 | 130.8 ± 85.0 | 3.99 ± 0.87 |
